# 

**Published:** 1897-07-31

**Authors:** 


### EARLY EDITION.

**Notices of Births, Deaths, and Marriages** are inserted in this column at a charge of 2s. 6d. for an announcement not exceeding four lines.

### NOTICE TO CORRESPONDENTS.

All MS., Letters, Books for Review, and other matters intended for the Editor should be addressed The Editor, "The Hospital," The Hospital Building, 28 & 29, Southampton Street, Strand, W.C.

The Editor cannot undertake to return rejected MS., even when accompanied by stamped directed envelope.

**Notice to Advertisers and Subscribers.**

All Advertisements, Orders for Copies of The Hospital, and Business Communications should be addressed to **THE MANAGER, (Not to The Editor),** The Hospital, The Hospital Building, 28 & 29, Southampton Street, Strand, London, W.C.

The Cost of Subscription, post free, is as follows:—Per week, 2½d.; per quarter, 2s. 8d.; per half-year, 5s. 3d.; per annum, 10s. 6d. Foreign:—Per annum, 15s. 2d.; payable, in all cases, in advance.

**NOTICE.—Vol. XXI. of "The Hospital" (October, 1896, to March, 1897), in handsome cloth binding, is on sale at the Office of this Journal, price 7s. 6d. Cases for binding the Numbers contained in this Volume, 1s. 6d. Index to Vol. XXI., 2½d., post free.**

**The Monthly Part for August will be ready on August 3rd. Price 6d., by post 8d.**

### BOOKS RECEIVED.

Burns and Oates.
"Memories of the Crimea." By Sister Mary Aloysius.

J. and A. Churchill.
"Convergent Strabismus and its Treatment." By Edwin Kotthouse.

Horace Marshall and Son.
"The London Manual for 1897-98."

Baillière, Tindall, and Cox.
"Franzenbad; Its Baths and Springs."

John Bale, Sons, and Danielson.
"The Journal of Balneology and Chinatology."

John Wright and Co.
"Diet Chart and Alimentary Index." By M. J. Kelly.

### The Student of Medicine.

### APPOINTMENTS.

A. S. Adams, M.B., C.M.Aberd., appointed Medical Officer for the Rillington District of the Norton Out Relief Union. A. Alexander, M.R.C.S., L.R.C.P.Lond., appointed Medical Officer for the Wellsbourne District of the Stratford-on Avon Union. J. D. Amenabar, M.R.C.S., L.R.C.P.Lond., appointed House Surgeon to the Wolverhampton Eye Infirmary. J. A. Anderson, M.D.Glasg., appointed Medical Officer to the Leswalt (Stranraer) Parish Council. G. Ashton, M.B., Ch.B.Vict., M.R.C.S.Eng., L.R.C.P.Lond., late House Surgeon, Manchester Southern Hospital, appointed Senior Resident Medical Officer to the Chorlton Union Hospitals, Withington, Manchester. T. Benson, L.R.C.P.Edin., M.R.C.S.Eng., appointed Medical Officer of Health to the Annfield Urban District Council. D. Brough, M.B., C.M.Edin., appointed House Surgeon to the Bridgwater Infirmary, Bridgwater. A. Cluckie, M.B., C.M.Glasg., appointed Medical Officer to the Stranraer Parish Council. P. Davidson, M.B., B.S.Durh., appointed Senior House Physician, Royal Infirmary, Newcastle on-Tyne. Dr. D. M. French, appointed Medical Officer for the Debenham District of the Bosmere and Claydon Union. W. Gosse, L.R.C.P.Lond., M.R.C.S., D.P.H.Camb., appointed Public Vaccinator to the Sittingbourne District of the Milton Union. J. Harrison, M.R.C.S., L.R.C.P.Edin., appointed Medical Officer in Charge of East Grinstead Isolation Hospitals. H. Hilliard, M.R.C.S.Eng., L.R.C.P.Lond., appointed Resident Medical Officer to St. George Hanover Square Dispensary.

